# Learning to detect sexism: An evaluation of the effects of a brief video-based intervention using ROC analysis

**DOI:** 10.3389/fpsyg.2022.1005633

**Published:** 2023-01-06

**Authors:** Regina König, Angela Heine

**Affiliations:** Department of Psychology, University of Duisburg-Essen, Essen, Germany

**Keywords:** sexism, intervention, signal detection theory, ROC, sensitivity, specificity

## Abstract

Empirical evidence for the effectiveness of interventions teaching lay people how to recognize sexism is scarce. The purpose of the present study was, thus, twofold: The first aim was to evaluate a brief intervention using a lecture-like educational video on how to recognize subtle sexism. The second aim was to demonstrate the usefulness of signal detection theory (SDT) for evaluating the participants’ ability to discriminate between subtle sexist and non-sexist statements. Participants (*N* = 73) were randomly assigned to a subtle sexism treatment group (SSG), an overt sexism treatment group (OSG), or a control group (CG). After the intervention phase, the participants were asked to rate statements in vignettes with respect to how sexist they perceived them to be. The participants in the SSG were significantly better in correctly identifying subtle sexist content than the participants in the OSG and CG. However, they were not more accurate overall. This was because they claimed sexism more often, irrespective of whether it was present or not. We conclude that while our intervention increased participants’ sensitivity in detecting sexist content, it did so at the cost of specificity. Our results make clear that practitioners teaching people how to recognize sexism should control intervention outcomes for unintended effects of biased decision criteria, given that erroneous allegations of sexism could have grave consequences. To this effect, the value of SDT, which allows for fine-grained and, consequently, more accurate insight than standard approaches to the analysis of intervention effects, was demonstrated.

## 1. Introduction

Sexism is a common problem with detrimental consequences for its targets (e.g., Swim et al., 2001; Leaper and Brown, 2014; Lawson, 2020). Despite this, there is a striking lack of systematic research on interventions to reduce sexism (Becker et al., 2014; Brown and Stone, 2016; Vescio and Kosakowska-Berezecka, 2020).

Being capable of recognizing sexist behavior and statements is of particular importance, since it is the first step in behavior change (Carnes et al., 2012; Pietri et al., 2017; Ashburn-Nardo and Karim, 2019). However, detecting sexism can be difficult because in modern egalitarian societies, the expression of blatant and hostile sexism is usually not tolerated. Therefore, sexist attitudes are generally expressed more covertly, e.g., disguised as humor or benevolent sexism (Barreto and Ellemers, 2013).

Ambivalent sexism theory (Glick and Fiske, 1996) contends that sexism is not necessarily expressed as antipathy and hostile attitudes, but also exists in the form of seemingly positive, benevolent attitudes. Benevolent and hostile sexism are complementary by ascribing positive or negative traits to men and women that are two sides of the same coin (Glick and Fiske, 1996, 1999, 2001). For instance, women are stereotyped as being warm but incompetent, and men as being cold but competent (Eckes, 2002). Although women are the structurally disadvantaged gender group and the main targets of sexism, it is important to also reduce biased attitudes against men (Becker et al., 2014). This is because ambivalent attitudes toward women, as measured by the *ambivalent sexism inventory* (ASI; Glick and Fiske, 1996), and ambivalent attitudes toward men, as measured by the *ambivalence toward men inventory* (AMI; Glick and Fiske, 1999), are positively correlated (Glick et al., 2004) and both reflect and reinforce the gender hierarchy and gender inequality (Haines et al., 2022).

Benevolent sexism (Swim et al., 2004; Barreto and Ellemers, 2013; Good et al., 2019) as well as non-prototypical forms of sexism, such as men being the targets of sexism (Ashburn-Nardo and Karim, 2019), are harder to detect. Critically, such subtle forms of sexism are similar or even more harmful than overt, more easily detectable forms of sexism (Jones et al., 2016). It is, thus, important to teach detection of not only overt, but also subtle sexism (Pietri et al., 2017; Ashburn-Nardo and Karim, 2019; Monteith et al., 2019).

To date, only a small number of studies have evaluated interventions aiming at increasing participants’ sensitivity in detecting sexism (Pietri et al., 2017; Ashburn-Nardo and Karim, 2019). Even fewer studies have taken into consideration whether increases in the correct detection of sexism are indeed due to an improved ability to discriminate between sexism and unbiased behavior. Alternatively, an increase of correct detection of sexism may be due to an overall tendency to perceive a person’s behavior as being sexist, regardless of whether or not it actually is. Two notable exceptions are the studies by Pahlke et al. (2014) and Pietri et al. (2017). Pietri et al. (2017) presented participants with scenarios that either constituted sexist content or not, to evaluate intervention effects. Participants of one of their two intervention groups were more confident in recognizing gender bias when it was present, but did not perform better in identifying the absence of bias compared to the control group. The authors concluded that their intervention increased sensitivity while not affecting specificity. Pahlke et al. (2014) trained children to recognize gender bias in media. They presented video clips reflecting sexism or, alternatively, counter-stereotypic behavior (a father doing housework) in a post-test and, again, in a follow-up. The authors reported that only one participant in the post-test and none of the participants in the follow-up test claimed sexism for the video without bias. They concluded that the intervention groups’ better performance in recognizing sexism when it was present “did not stem from indiscriminate claims of gender bias” (p. 127). However, they reported perfect performance in the condition without sexism which could indicate a ceiling effect (Hautus et al., 2022), i.e., problems with validity.

A major methodological problem of such approaches to the analysis of intervention effects is that considering sensitivity in isolation falls short of capturing discrimination performance accurately. This is because sensitivity and specificity are both indicators for accuracy in need to be taken into account (Herzog et al., 2019). Sensitivity, the rate of correctly identified instances of sexism, represents accuracy when sexism is present. Specificity, the rate of correctly identified instances of the absence of sexism represents accuracy when sexism is not present (Metz, 1978; Herzog et al., 2019). Signal detection theory (SDT) provides a methodological approach to examine sensitivity and specificity in combination to gauge performance. The assumption underlying SDT is that people make decisions under uncertainty, e.g., due to a lack of or ambiguity of information provided. SDT is suitable to measure accuracy in deciding whether a relevant target is present (*signal*) or absent (*noise*). The more unequivocally perceivable the signal is as compared to the noise, the less the perceiver is faced with perceptual overlap between signal and noise. Less perceptual overlap, in turn, results in higher accuracy (Anderson, 2015). To make a decision despite uncertainty, the decision-maker has to adopt one or more decision criteria that separate signal from noise (Pastore and Scheirer, 1974).

Whether and under which circumstances people tend to underreport or overreport experiences of prejudice, such as sexism, has been a topic of ongoing debate. It can be difficult to recognize a sexist event as sexist. On the other hand, non-discriminatory actions may be misperceived as sexist under certain circumstances (Barreto and Ellemers, 2015). Correct detection of sexism after intervention can be driven by higher ability to discriminate between sexist and non-sexist content or, alternatively, by a tendency to overreport sexism, i.e., response bias. Correct detection of the absence of sexism can be driven by higher ability to discriminate, or by a tendency to underreport sexism (Greenwald et al., 2003).

SDT allows for differentiating between accuracy in detection on the one hand, and response bias on the other hand. People can be biased toward a *liberal decision criterion*, i.e., a bias toward reporting sexism, or toward a *conservative decision criterion*, i.e., a bias toward reporting absence of sexism (Anderson, 2015). A liberal criterion results in higher sensitivity, but also in lower specificity. In contrast, a conservative criterion leads to lower sensitivity and higher specificity (Hautus et al., 2022). SDT is especially suited for research on the detection of sexism because people’s evaluations of events are likely to be influenced by response bias. For instance, people with stronger belief in meritocracy are less likely to perceive prejudice (Barreto and Ellemers, 2015). Hence, it is of theoretical and practical importance to differentiate between accuracy and response bias.

In SDT, receiver operating characteristic (ROC) curves are used to represent the relationship between a participant’s hit and false alarm rates at different decision criteria. ROC curves can be used to characterize accuracy when a participant is given the opportunity to not only decide whether sexism is present or not, but also to rate the degree of confidence (Hautus et al., 2022). When using, for instance, a scale from 1 (*definitely not sexist*) to 5 (*definitely sexist*), the participant needs to adopt 5–1 = 4 decision criteria to decide between adjacent pairs of responses. The resulting ROC curve represents the participant’s detection accuracy at all possible decision criteria (Pastore and Scheirer, 1974). The area under the curve (AUC) measures the area that lies underneath the ROC curve. When accuracy in discriminating sexist from non-sexist cases is at chance level, AUC = 0.05. Perfect accuracy would result in AUC = 1.0. In the present study, the AUC represents the probability that a participant rates any sexist vignette as more likely to be sexist than any non-sexist vignette (Kumar and Indrayan, 2011).

### 1.1. The present study

To illustrate the utility of SDT in evaluating the ability to detect subtle sexism, the present study will test the effectiveness of a brief educational video on how to detect subtle sexism. We will contrast the results of standard group comparisons of ratings of sexist and non-sexist vignettes with an SDT-based analysis.

Using a standard statistical approach, mixed repeated-measures analysis of variance (ANOVA), we will test the following hypotheses:

*Hypothesis 1*: The participants in the subtle sexism group (SSG) will rate subtle sexist vignettes as significantly more sexist than the participants in an overt sexism group (OSG) and a control group (CG). Thus, Hypothesis 1 predicts higher sensitivity in the SSG as compared to the OSG and CG.

*Hypothesis 2*: The participants in the SSG will rate non-sexist vignettes as significantly less sexist than the participants in the OSG and CG. Hypothesis 2, thus, predicts higher specificity in the SSG as compared to the OSG and CG.

SDT allows us to formulate an alternative, more elaborate, combined hypothesis:

Composite hypothesis:

Participants in the SSG will show a significantly larger AUC as compared to the participants in the OSG and CG. The composite hypothesis, thus, predicts higher detection accuracy in the SSG as compared to the OSG and CG on a single, overall measure that takes into account not only sensitivity and specificity in combination, but also response bias.

## 2. Materials and methods

### 2.1. Participants

An *a priori* power analysis using g*power (Faul et al., 2009) was done, assuming a small to medium effect size of partial *η^2^* = 0.035 (Pallant, 2020) as outcome of a within-between ANOVA with three groups (between-subjects factor) and two measures (ratings of the sexist and the non-sexist vignettes; within-subjects factor). A total sample size of 72 participants was recommended to reach statistical power of 1–*β* = 0.80, given an *α* = 0.05.

The sample consisted of *N* = 73 adult participants. Due to a technical error during data collection, the variable age had to be recoded into categories. Of the 73 participants, 35 (47.9%) were aged between 18 and 25 years, 19 (26.0%) were aged between 26 and 35 years, 9 (12.3%) between 36 and 45 years, and 10 (13.7%) were aged between 46 and 85 years. Twenty-one (28.8%) participants self-identified as male, 51 (69.9%) as female, and one (1.4%) participant as diverse. According to German law, diverse refers to non-binary or a gender identities. Of the 73 participants, 20 (27.4%) stated to have an academic degree, 13 (17.8%) stated to have a non-academic, professional qualification, 36 (49.3%) were university students, and three (4.1%) stated to currently receive vocational training. One participant (1.4%) indicated to neither receive nor having completed any vocational training. Participants were invited to take part in a study about civic engagement and assigned randomly to one of three experimental groups.

The study was approved by the local ethics committee at the University of Duisburg-Essen. All data were collected anonymously after the participants had provided their informed consent.

### 2.2. Materials

#### 2.2.1. Development of vignettes

We developed and pre-tested 24 short vignettes to measure participant’s ability to detect sexism. Further details regarding the pre-test are outlined in the Supplementary material. The eight sexist vignettes were developed based on the ASI (Glick and Fiske, 1996) and the AMI (Glick and Fiske, 1999). The sexist vignettes included either prototypical benevolent sexist statements, non-prototypical benevolent sexist statements, or hostile but non-prototypical (e.g., hostile sexism against men, women as perpetrators of sexism) sexist statements which are more difficult to detect than hostile and prototypical forms of sexism (Swim et al., 2004; Barreto and Ellemers, 2013; Ashburn-Nardo and Karim, 2019; Good et al., 2019). Eight non-sexist vignettes included statements that were not sexist but related to a man or a woman. In addition, eight neutral vignettes containing statements that were not related to gender were used as fillers. All vignettes that were used in the present study are listed in the Supplementary materials.

#### 2.2.2. Training material

For the intervention, we created a short video of approximately 16 min in line with the definition of sexism as “attitudes, beliefs, or behaviors that support the unequal status of women and men” (Swim and Campbell, 2003, p. 219). The content of the video complied with Brown (2011) who suggests that effective interventions in this domain should include “an explicit discussion of discrimination, stereotypes, and exclusion [and] an explicit discussion of the ways in which stereotypes are inaccurate” (pp. 3–4). The first part of the video started with an introduction of sexism as comprising gender stereotypes, prejudice, and discrimination. This was followed by explanations on the content of gender stereotypes (Fiske, 2015). A brief discussion of empirical findings on the accuracy of stereotypes followed (Jussim et al., 2009; Helgeson, 2012). It was emphasized that men and women have far more similarities than dissimilarities (Hyde, 2005). In the second part of the video, the concepts of hostile and benevolent sexism (Glick and Fiske, 1996) were introduced and illustrated with examples of hostile and benevolent attitudes toward women and men. In addition, the concept of implicit gender bias (Greenwald and Krieger, 2006; Eckes, 2010) was introduced. The video ended with a brief discussion of costs and benefits of confronting sexism. Potential consequences of not confronting sexist bias were pointed out, and everyone’s responsibility to act against sexism was emphasized (Czopp, 2019).

#### 2.2.3. Control treatments

The video presented to the OSG was based on information on sexism and how to confront it provided by the German Agency for Civic Education (Bundeszentrale für politische Bildung (bpb), n.d.-b). It was approximately 13 min in length. In contrast to the video on subtle sexism, the video presented to the overt sexism group did not focus on subtle sexism but on more overt forms of sexism, such as sexual harassment. In this video, sexism was defined as personal and structural discrimination based on gender. It was pointed out that every gender can be the target of sexism but that mostly women are affected by it.

Approximately 17 min long video for the CG focused on conspiracy theories and how to confront them. The content was based on Bundeszentrale für politische Bildung (bpb) (n.d.-a) and on Douglas et al. (2019).

#### 2.2.4. Procedure and analysis

From the 24 vignettes, we selected the six sexist and six non-sexist vignettes that were most unequivocally judged as sexist and non-sexist, respectively, by the expert raters. After watching the videos, all participants in all experimental groups were presented with the sexist, non-sexist, and the eight neutral vignettes in random order. The participants were asked to indicate how sexist they perceived the statement in each vignette to be on a 7-point Likert scale (*cf.* Table 1). An odd-numbered scale was chosen to provide for one response option in case of uncertainty. Scale means were computed by averaging the participants’ ratings across the vignettes within each category of vignettes. Higher ratings of the sexist vignettes as sexist indicate higher sensitivity; lower ratings of the non-sexist vignettes indicate higher specificity.

**Table 1:** Frequencies of responses to the statement: this statement is sexist.

	1 do not agree at all	2 do not agree	3 do rather not agree	4 cannot decide	5 do rather agree	6 do agree	7 do completely agree	Total
	**Subtle sexism group** (*n* = 26)							
Non-sexist vignettes	39	27	22	6	23	20	19	156
	False alarm rate^a^: 0.397							
Sexist vignettes	3	4	1	11	24	47	66	156
	Hit rate^b^: 0.878							
	**Overt sexism group** (*n* = 24)							
Non-sexist vignettes	38	14	18	12	29	19	14	144
	False alarm rate^a^: 0.431							
Sexist vignettes	13	7	8	9	24	38	45	144
	Hit rate^b^: 0.743							
	**Control group** (*n* = 23)							
Non-sexist vignettes	58	18	14	6	18	16	8	138
	False alarm rate^a^: 0.304							
Sexist vignettes	15	14	13	5	32	27	32	138
	Hit rate^b^: 0.659							

a The false alarm rate was computed as sum of ratings from 5 to 7 of the non-sexist vignettes divided by the sum of all ratings of the non-sexist vignettes.

b The hit rate was computed as sum of ratings from 5 to 7 of the sexist vignettes divided by the sum of all ratings of the sexist vignettes.

In order to make the main purpose of the study less obvious, and to make the study appear more plausible for participants in the CG, participants in all experimental groups completed the *vaccine conspiracy beliefs scale* (Shapiro et al., 2016) before watching the video. Given that the capacity to detect sexism is negatively correlated with people’s own sexist attitudes (Cameron, 2001; Swim et al., 2004), all participants were presented with the *neosexism scale* (Campbell et al., 1997; German translation by Blanz, 1999) at the beginning of the study to control for sexist attitudes. After rating the vignettes, all participants were presented with other questionnaires for exploratory purposes only, i.e., the generic conspiracist beliefs scale (Brotherton et al., 2013); the ambivalent sexism scale (Eckes and Six-Materna, 1999); and the perceived vulnerability to sexism scale (Weis et al., 2018). These questionnaires were unrelated to the hypotheses of the present study, and will not be considered any further.

For the present study, we employed multi-reader multi-case (MRMC) analysis (Gallas, 2006) to estimate AUCs. MRMC can be used to analyze a fully crossed design where all readers (i.e., participants) evaluate all cases (i.e., stimuli) in all (experimental) conditions, i.e., groups. A prototypical use case for this approach would be when a group of radiologists evaluates images of sick and healthy patients one time using a certain computer-aided diagnostic support system and a second time without any aids (Gallas and Brown, 2008). To accommodate a larger range of study approaches, an extension of MRMC was implemented which can handle designs that are not fully crossed (Gallas, 2006; Gallas and Brown, 2008). One type of a non-fully crossed design is the *unpaired-reader paired case* (Gallas and Brown, 2008), which is related conceptually to a mixed repeated-measures design where, for instance, one group of radiologists evaluates images of healthy and sick individuals (within-subjects factor) with the help of a support system, while a second group evaluates the same images without using this system (between-subjects factor). In general terms, this extended MRMC approach can handle designs where different groups of participants rate the same stimuli under different experimental conditions. In the present study, *unpaired reader* corresponds to participants being randomly assigned to one of the three experimental groups. In each of the groups, participants were presented with one of the three educational videos. The random assignment to the experimental groups represents the between-subjects factor. *Paired case* refers to all participants being asked to rate all of the sexist, non-sexist, and neutral vignettes. The sexist and non-sexist vignettes represent the within-subjects factor.

There were no missing data and no significant outliers. Thus, the data of all *N* = 73 participants were included in the analyses. The ANOVA models were estimated with SPSS version 28. The MRMC analyses were conducted using the software iMRMC, Version V4.0.3 (Gallas, 2006; Gallas et al., 2009).

## 3. Results

The SSG (*M* = 1.89; *SD* = 0.68), OSG (*M* = 1.88; *SD* = 0.61), and CG (*M* = 1.94; *SD* = 0.79) did not differ regarding their neosexist attitudes, *F* (2, 70) = 0.05, *p* = 0.951, partial *η^2^* = 0.01.

The mean ratings of the sexist, non-sexist, and neutral vignettes are shown in Table 2. Two of the neutral vignettes had item-scale correlations below.3, resulting in an insufficient scale reliability of *α* = 0.639. Thus, these two vignettes were removed. The neutral vignettes scale with six items had an acceptable reliability, *α* = 0.707. Given that participants in all groups rated the neutral vignettes as not sexist, *F* (2, 70) = 1.34, *p* = 0.268, partial *η^2^* = 0.04, the neutral vignettes were excluded from further analyses.

**Table 2:** Descriptive statistics of the mean ratings of the six sexist, the six non-sexist, and the six neutral vignettes across the experimental groups.

	Subtle sexism group (*n* = 26)	Overt sexism group (*n* = 24)	Control group (*n* = 23)	Total (*N* = 77)
Vignettes^a^	*M (SD)*	*M (SD)*	*M (SD)*	*M (SD)*
Sexist vignettes: “The statement is sexist.” *α* = 0.847	5.91 (0.88)	5.21 (1.37)	4.84 (1.47)	5.34 (1.32)
Non-sexist vignettes: “The statement is sexist.” *α* = 0.755	3.53 (1.06)	3.65 (1.37)	2.91 (1.42)	3.37 (1.31)
Neutral vignettes: “The statement is sexist.” *α* = 0.707	1.44 (0.59)	1.34 (0.48)	1.21 (0.38)	1.34 (0.50)

a The participants responded on Likert-type scales from 1 (strongly disagree) to 7 (strongly agree). The items were presented in German.

Mixed repeated-measures analyses of variance (ANOVAs) with *type of vignette* (sexist, non-sexist) as within-subject factor, and *group* (TG, CG1, CG2) as between-subjects factor resulted in a main effect of *type of vignette*, *F* (1, 70) = 176,74, *p* < 0.001, partial *η^2^* = 0.72. Across all groups, participants rated the sexist vignettes as substantially more sexist than the non-sexist vignettes. The interaction between *type of vignette* and *group* did not reach conventional levels of statistical significance, *F* (2, 70) = 2.65, *p* = 0.078, partial *η^2^* = 0.07.

There was a medium to large effect of *group* on participants’ ratings of the vignettes, *F* (2, 70) = 3.63, *p* = 0.032, partial *η^2^* = 0.09. Simple effects analyses with planned comparisons revealed that the SSG rated the sexist vignettes as significantly more sexist than the OSG and CG, *F* (1, 70) = 8.33, *p* = 0.005, *d* = 1.41, supporting Hypothesis 1. This difference corresponds to a very large effect. Simple effects analyses with planned comparisons revealed that in identifying the non-sexist vignettes, the three experimental groups performed equally, *F* (1, 70) = 0.65, *p* = 0.424, *d* = 0.39. Hypothesis 1 was, thus, not supported.

**Figure 1:**
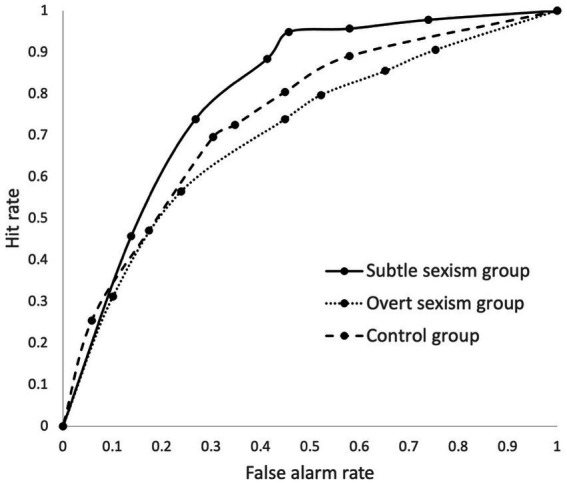
Pooled average ROC curves of the experimental groups.

On a descriptive level, the frequencies of ratings of the sexist and non-sexist vignettes (*cf.* Table 1) indicate that the SSG performed best regarding the correct detection of the sexist vignettes. Unexpectedly, though, the participants in the SSG and OSG showed a higher false alarm rate than the participants in the CG (*cf.* Table 1). Figure 1 shows that the participants in the SSG tended to perform better at nearly all threshold levels, except at levels of very low false alarm rates. However, this difference was not significant. Neither the difference between the AUCs of the SSG (*AUC* = 0.802, *SE* = 0.065) and OSG (*AUC* = 0.758, *SE* = 0.074) nor the difference between the AUCs of the SSG and the CG (*AUC* = 0.761, *SE* = 0.072) were significant (*p* = 0.336, 95% CI [−0.054, 0.133], and *p* = 0.472, 95% CI [−0.071, 0.153], respectively). The composite hypothesis was, thus, not supported.

## 4. Discussion

The present study aimed at the evaluation of the effectiveness of a brief intervention to recognize subtle forms of sexism such as benevolent and non-prototypical sexism. Looking into participants’ ratings of sexist and non-sexist vignettes as separate measures for performance change suggests that the intervention was indeed effective in increasing participants’ performance in detecting subtle sexism. As hypothesized, participants in the SSG were better in detecting sexism than the participants in the OSG and CG. At the same time, the identification of non-sexist vignettes did not differ between the experimental groups. So far, our results are in line with the findings of Pietri et al. (2017).

However, further analyses using SDT provided a more fine-grained and considerably less equivocal interpretation of the effects of our intervention. Based on the ROC analysis, participants in the SSG were actually not more accurate in discriminating sexist from non-sexist content as compared to the participants in the OSG and CG. Even though the intervention resulted in increased numbers of correctly identified sexist vignettes, it came at the cost of a concomitant increase of false alarms. The intervention did, thus, not improve participants’ ability to discriminate between sexist and non-sexist content, but rather resulted in participants claiming sexism more often, regardless of whether it was present or not.

Given that the educational videos used for the SSG and for the OSG were implemented on the basis of standard approaches to reduce sexism (Brown, 2011; Bundeszentrale für politische Bildung (bpb), n.d.-b), the problem of unintended effects in terms of losses in specificity is not only of theoretical, but also of practical relevance. Reduced specificity may result in erroneous allegations of sexism which may have serious consequences. Recent research found, e.g., that there are rising concerns in society regarding erroneous allegations of sexism (Bhattacharya and Stockdale, 2016; Atwater et al., 2019). Fear of erroneous allegations, in turn, may harm the career opportunities of young women (Soklaridis et al., 2018).

The main limitation of the current study is that we only looked into intervention effects immediately after the participants watched the videos, even though testing for short-term effects is a common practice in research aiming to reduce sexism (Bigler and Pahlke, 2019) in particular, as well as in the literature on prejudice reduction (Paluck et al., 2021) in general. It remains unclear what the intervention effects would have been over a longer period of time. Additionally, this type of short-term intervention may not be suitable to impart sufficient knowledge regarding the complex issue of sexism (Paluck, 2012; Bigler and Pahlke, 2019; Paluck et al., 2021). Indeed, past research has found that brief, single interventions aimed at reducing intergroup biases tend to be of limited benefit only (FitzGerald et al., 2019; Paluck et al., 2021). Therefore, future studies should look into the issue of whether and how brief intervention modules like the present one can be integrated into more comprehensive, long-term intervention schemes (Chang et al., 2019).

## 5. Conclusion

The present study demonstrates that brief interventions helping people to recognize sexism can, indeed, improve sensitivity to sexism. Our intervention did, however, not have the intended effect on participants’ overall ability to discriminate between sexist and non-sexist content. Participants who watched educational videos on sexism tended to claim sexism not only in sexist but also in non-sexist vignettes. Educators and practitioners in the field should, thus, not only focus on improving sensitivity for sexism but also on avoiding losses in specificity due to biased decision criteria. Furthermore, the present study demonstrated that SDT can be a valuable tool for a fine-grained evaluation of the effectiveness of interventions in this domain. SDT provided for more accurate conclusions than the more common approach to analysis that considers ratings of sexist and non-sexist vignettes as separate outcomes and neglects the impact of possible response biases.

## Data availability statement

The data sets presented in this study can be found in online repositories. The names of the repository/repositories and accession number(s) can be found at: Data set SPSS: https://doi.org/10.6084/m9.figshare.20375058; Codebook for data set SPSS: https://doi.org/10.6084/m9.figshare.20375139; Syntax for data analysis in SPSS: https://doi.org/10.6084/m9.figshare.20391693; Data set iMRMC: https://doi.org/10.6084/m9.figshare.20375184; Codebook for data set iMRMC: https://doi.org/10.6084/m9.figshare.20375166; Readme for the data analysis performed in iMRMC: https://doi.org/10.6084/m9.figshare.20375193; Materials: https://doi.org/10.6084/m9.figshare.20375781. All files are stored at Figshare.

## Ethics statement

The studies involving human participants were reviewed and approved by the Ethics Committee of the Department of Psychology, University of Duisburg-Essen, Faculty of Educational Sciences, Department of Psychology. The patients/participants provided their written informed consent to participate in this study.

## Author contributions

All authors listed have made a substantial, direct, and intellectual contribution to the work and approved it for publication.

## Conflict of interest

The authors declare that the research was conducted in the absence of any commercial or financial relationships that could be construed as a potential conflict of interest.

## Publisher’s note

All claims expressed in this article are solely those of the authors and do not necessarily represent those of their affiliated organizations, or those of the publisher, the editors and the reviewers. Any product that may be evaluated in this article, or claim that may be made by its manufacturer, is not guaranteed or endorsed by the publisher.
