# Supplementary material for: Learning to detect sexism: An evaluation of the effects of a brief video-based intervention using ROC analysis
Source: Front Psychol. 2023 Jan 6;13:1005633. doi: 10.3389/fpsyg.2022.1005633 (PMC9853915; doi:10.3389/fpsyg.2022.1005633)
Supplement: Supplementary file 1 [file Data_Sheet_1.PDF]

Supplementary Materials for *Learning to detect sexism: An evaluation of the effects of a brief video-based intervention using ROC analysis*

Pre-test of the vignettes

In a first step, independent expert raters ($N = 8$) employed at the department of psychology of the University of Duisburg-Essen rated the vignettes with regard to how sexist they perceived them to be and regarding the subtlety of the sexist statements. Each expert rated 12 out of the 24 vignettes ($N = 4$ per vignette).

The four experts in group 1 rated the first subset of sexist vignettes ($M = 5.19$, $SD = 0.46$) as significantly more sexist than the first subset of non-sexist vignettes ($M = 3.79$, $SD = 0.47$; $t(3) = 3.42$, $p = .021$) on a Likert-type scale from 1 (*not sexist at all*) to 6 (*very sexist*). Likewise, the four experts in group 2 rated the second subset of sexist vignettes ($M = 4.96$, $SD = .42$) as significantly more sexist than the second subset of non-sexist vignettes ($M = 2.78$, $SD = .84$; $t(3) = 7.49$, $p = .002$).

For the present study, we selected the six sexist and six non-sexist vignettes that were most unequivocally rated by the experts. All vignettes that were used in the present study are listed in Table S1.

Table S1: Wording of the sexist, non-sexist, and neutral vignettes

Vignette label	Vignette: Original wording	Vignette: English translation	Commentary
		**Sexist vignettes**	
V2_s	Alexander ist seit Kurzem Erzieher in einem Waldkindergarten. Seine Vorgesetzte informiert ihn: "Sie werden in eine andere Gruppe versetzt. Die Mutter eines der Kinder in Ihrer Gruppe hat kein gutes Gefühl bei einem Erzieher."	Alexander has recently started working as a nursery schoolteacher. His boss tells him: „You will be placed in another class of the kindergarten. The mother of one child in your current class feels uncomfortable with a male nursery schoolteacher."	Non-prototypical (woman against man) and hostile (heterosexual hostility due to women's awareness of threats of sexual violence by men (Glick & Fiske, 1999))
V8_s	Carina weint sich bei ihrem Bruder Niklas über die Probleme in ihrer Beziehung aus. Niklas findet: „Du solltest ihn verlassen. Wenn ein Mann seine Frau nicht auf Händen trägt, ist er den Ärger nicht wert."	Carina is meeting her brother Niklas and is complaining about the problems in her relationship. Niklas argues: „You should dump him. When a men doesn't dance attendance on his wife, he is not worth the trouble."	Prototypical (man against women) and benevolent (protective paternalism (Glick & Fiske, 1996))
V9_s	Julia, Maja und Klara suchen eine vierte Person für ihre WG. Nachdem sich einige Personen vorgestellt haben, diskutieren die drei, wen sie auswählen möchten. Julia argumentiert: „Ich würde Niklas nehmen. Ein Mann bringt Gelassenheit und Ruhe in eine Frauengruppe."	Julia, Maja, and Klara are looking for a fourth flatmate. After some people have applied for the room, the three of them discuss whom to choose. Julia says: „I would choose Niklas. A man brings calm and serenity into a group of women."	Non-prototypical (women against women) and hostile (ascribing bitchiness to women (Glick & Fiske, 1996))
V17_s	Heiko und sein Mitschüler Tilo unterhalten sich über ihre Berufswünsche. Heiko	Heiko and his fellow student Tilo are talking about their career aspirations. Heiko says: „I	Non-protoypcial (man against men) and benevolent (complementary

	sagt: „Ich möchte einen Job, der zu mir als Mann passt. Ich will geistig und körperlich gefordert werden.“	want a job that suits me as a man. I want an intellectual and physical challenge.“	gender differentiation; ascribing men instrumental competence and physical strength (Glick & Fiske, 1999))
V18_s	Michael und Ulf unterhalten sich über Beziehungen und Heirat. Ulf witzelt: „Wenn wir die Frauen nicht hätten, würden wir Männer uns vermutlich ausschließlich von Tiefkühlpizza und Bier ernähren.“	Michael and Ulf are talking about relationships and marriage. Ulf says jokingly: „I we wouldn’t have women, we as men would probably only exist on a diet of frozen pizza and beer.“	Non-prototypical (men against men) and hostile (maternalism; male weakness requiring female care (Glick & Fiske, 1999))
V20_s	Astrid und Birgit sitzen zusammen bei Kaffee und Kuchen. Birgit erzählt, dass sie kürzlich mit ihrem Freund zusammengezogen ist und sagt: „Er ist ein richtiger Macher. Endlich habe ich einen Mann bei mir im Haus.“	Astrid and Birgit are meeting for coffee and cake. Birgit shares that she has recently moved in with her boyfriend and says: „He is a man of action. Finally, I have now a man in the house.“	Non-prototypical (woman against man) and benevolent (complementary gender differentiation; ascribing men instrumental competence physical strength (Glick & Fiske, 1999))
		**Non-sexist vignettes**	
V4_ns	Kilian und Lukas waren Paintball spielen mit ihrer neuen Mitbewohnerin Luise. Am nächsten Tag meint Lukas zu Kilian: „Wahnsinn, ich war wirklich beeindruckt, dass sie so gut schießen kann.“	Kilian and Lukas went playing paintball with their new flatmate Luise. The next day Lukas says to Kilian: „Wow, I was really impressed by her shooting skills.“	
V7_ns	Henrik, Ralph und Viktoria überlegen, was sie ihrer Kollegin zum ihrem Geburtstag schenken könnten. Henrik sagt: „Wie wäre es mit	Henrik, Ralph and Viktoria are discussing about which birthday present they could give to their colleague. Henrik	

	einem Kuchen? Viktoria, du kannst doch gut backen.“	says: „How about a cake? Viktoria, you make amazing cakes, don’t you?“
V11_ns	Karl und Detlef schrauben abends zusammen an einem Oldtimer. Karl reicht Detlef versehentlich ein falsches Werkzeug und Detlef spöttelt: „Mensch Karl, du solltest wenigstens einen Schraubendreher von einem Inbusschlüssel unterscheiden können."	Karl and Detlef are working together on a vintage car. Karls hands Detlef the wrong tool by accident and Detlef says jokingly: „Good grief, Karl, you should at least be able to distinguish a screwdriver from a hex key.“
V12_ns	Karen und Martina unterhalten sich über ihre Kollegin Ute. Ute klagte in letzter Zeit öfter mehrfach über eine zu hohe Stressbelastung an ihrem Arbeitsplatz. Martina kommentiert: „Die Frau ist einfach zu weinerlich.“	Karen and Martina are talking about their colleague Ute. Ute has been complaining for some time about being too stressed at work. Martina says: „This woman is too whiny.“
V13_ns	Nina und Martin sind frischgebackene Eltern. Einer Freundin erzählt Nina: „Martin ist wirklich ein toller Vater. Meinen Mann kann zum Glück einfach nichts aus der Ruhe bringen.“	Nina and Martin have recently become parents. Nina tells one of her friends: „Martin is a fantastic father. My husband has got nerves of steel.“
V15_ns	Zur Firmenweihnachtsfeier hat Torsten einen Kuchen mitgebracht. Frieda ist total begeistert und sagt in die Runde: „Der Kuchen ist wahnsinnig gut. Hat Torsten selbst gebacken.“	Torsten has brought a cake to the company’s Christmas party. Frieda is delighted and says to all: „The cake tastes great. It is self-made.“
	**Neutral vignettes**	

V1_n	Dagmar nimmt sich für das neue Jahr vor, eine bessere Work-Life-Balance für sich zu finden. Sie sagt zu ihrer besten Freundin: „Ein Workshop für Meditation und Achtsamkeit würde uns beiden gut tun. Bist Du dabei?“	Dagmar’s new year’s resolution is to engage in a better work life balance. She asks her best friend: „The both of us could use a workshop for mediation and mindfullness. Would you like to join me?“	Removed from scale due to low item-total correlation
V3_n	Florian, Lisa und Fatma wollen gemeinsam einen Film im Kino sehen und danach noch etwas trinken gehen. Im Kino können sie sich jedoch nicht auf einen Film einigen und Florian sagt: „Lasst uns das mit dem Film seinlassen und einfach sofort in eine Bar gehen.“	Florian, Lisa, and Fatma plan on going to the movies and to have a drink together afterwards. At the cinema, however, they cannot decide which movie to watch and Florian suggests: „Let’s skip the plan to watch a movie and let’s go directly to the bar.“	
V5_n	Jochen interessiert sich sehr für Malerei, kann dieses Interesse in seinem Freundeskreis aber leider nicht teilen. Spontan fragt er seinen neuen Kollegen: „Hättest du vielleicht Lust, mit mir am Sonntagnachmittag in's Kunstmuseum zu gehen? Es gibt dort eine neue Ausstellung.“	Jochen is very much interested in painting. His friends, however, don’t share his passion. He spontaneously asks his new colleague: „Would you like to join me for a visit in the arts museum on Sunday afternoon? They have got a new exhibition.“	
V6_n	Günther fühlt sich jetzt, einige Monate nach seiner Scheidung, immer einsamer. Sein Bruder rät ihm, sich ein Hobby zu suchen. Er schlägt vor: „Melde Dich doch beim örtlichen Skatclub an! Dort findest Du sicher neue Kumpels."	Günther has fehlt more and more lonely since his divorce. His brother recommends finding a new hobby. He says: „You should enroll for Skat session in the local club. You will be able to find new friends there for sure.“	Removed from scale due to low item-total correlation

V10_n	Gisela ist für ihren neuen Job in eine andere Stadt gezogen. Sie klingelt bei ihrer neuen Nachbarin, stellt sich vor und sagt: „Ich habe mich gefragt, ob Sie vielleicht Lust hätten, mir bei Gelegenheit bei einem Spaziergang die Umgebung zu zeigen."	Gisela has moved to another city for her new job. She is ringing the doorbell of her new neighbor asking her: „I wondered wether you would like to show me the neighborhood some time."
V14_n	Murat möchte gerne ehrenamtlich arbeiten, ist aber unsicher, was er tun könnte. Ein Kollege schlägt vor: „Ich kenne eine Initiative, die Vorlesestunden für Grundschulkinder anbietet. Bewirb dich doch da!"	Murat would like to volunteer in his free time but is unsure which task could suit him. A colleague of him is suggesting: „Ich know a place that offers story times for children. You should offer your help there!"
V16_n	Dieter ist seit einiger Zeit unglücklich mit seinem Job. Er versteht nicht recht, was mit ihm selbst los ist. Er erklärt seinem Kumpel: „Damals im Studium war dieser Job noch genau das, was ich machen wollte."	Dieter has been unhappy in his job for some time now. He doesn't really now what's wrong with him. He tells his friend: „When I was a student, this was the job of my dreams."
V19_n	Alexandra und ihr Mann gehen durch den Supermarkt und sie merkt plötzlich, wie ihr der Magen knurrt. Sie sagt: „Ich habe Lust auf Tiefkühlpizza. Dabei hatte ich mir doch fest vorgenommen, mich gesünder zu ernähren."	Alexandra and her husband are walking through the supermarket when she starts to feel hungry. She says: „I would fancy a frozen pizza although I had planned to eat more healthy now."

Literature

Glick, P., & Fiske, S. T. (1996). The ambivalent sexism inventory: Differentiating hostile and benevolent sexism. *Journal of Personality and Social Psychology*, *70*, 491-512. https://doi.org/10.1037/0022-3514.70.3.491

Glick, P., & Fiske, S. T. (1999). The ambivalence toward men inventory: Differentiating hostile and benevolent beliefs about men. *Psychology of Women Quarterly*, *23*(3), 519-536. https://doi.org/10.1111/j.1471-6402.1999.tb00379.x
